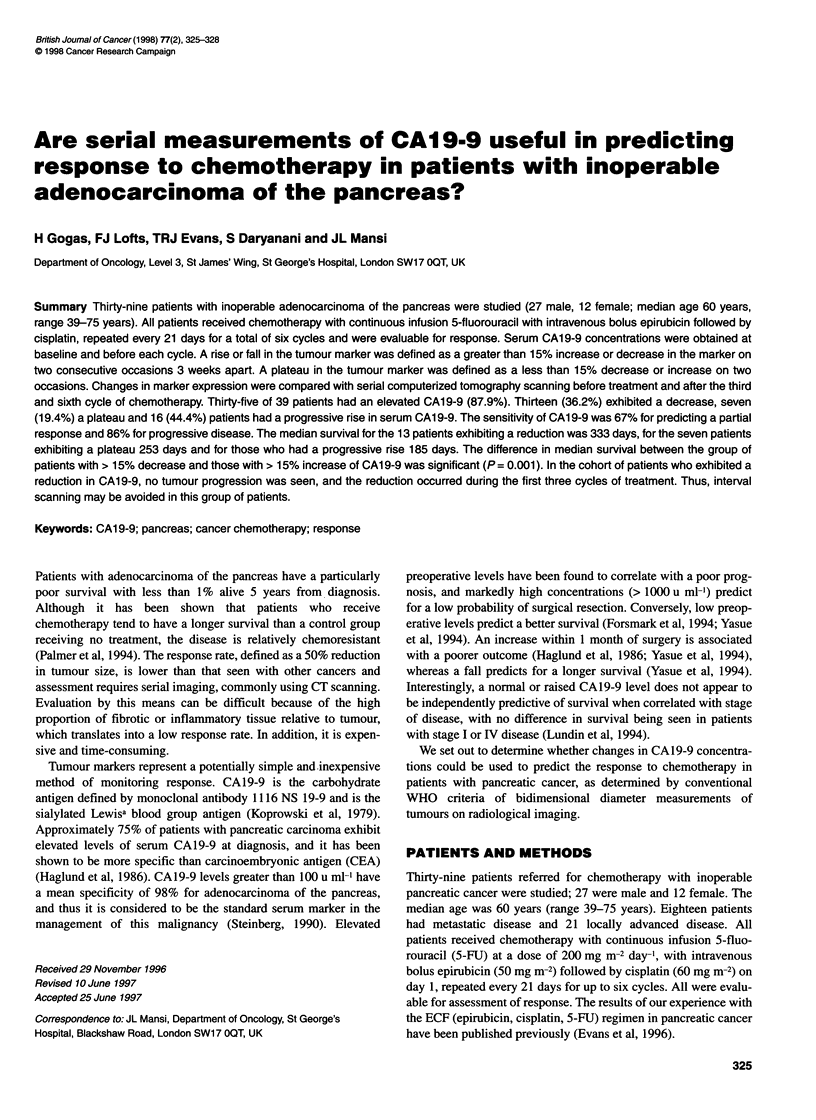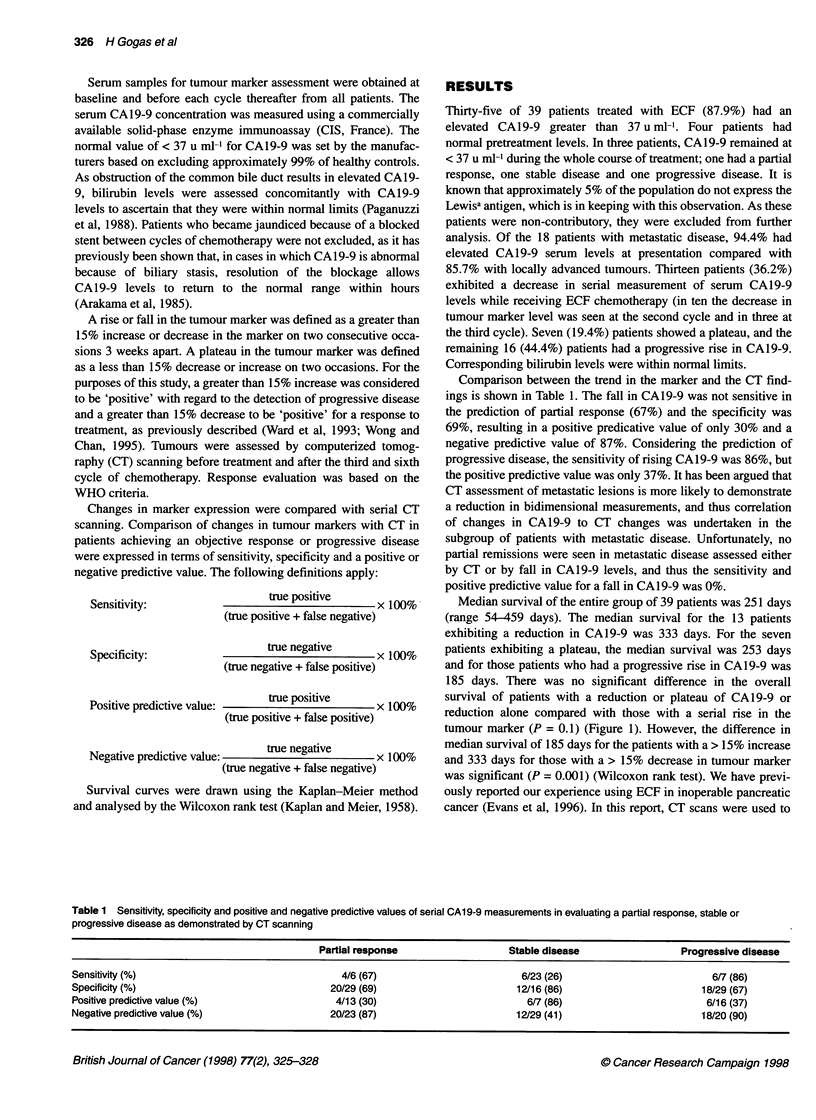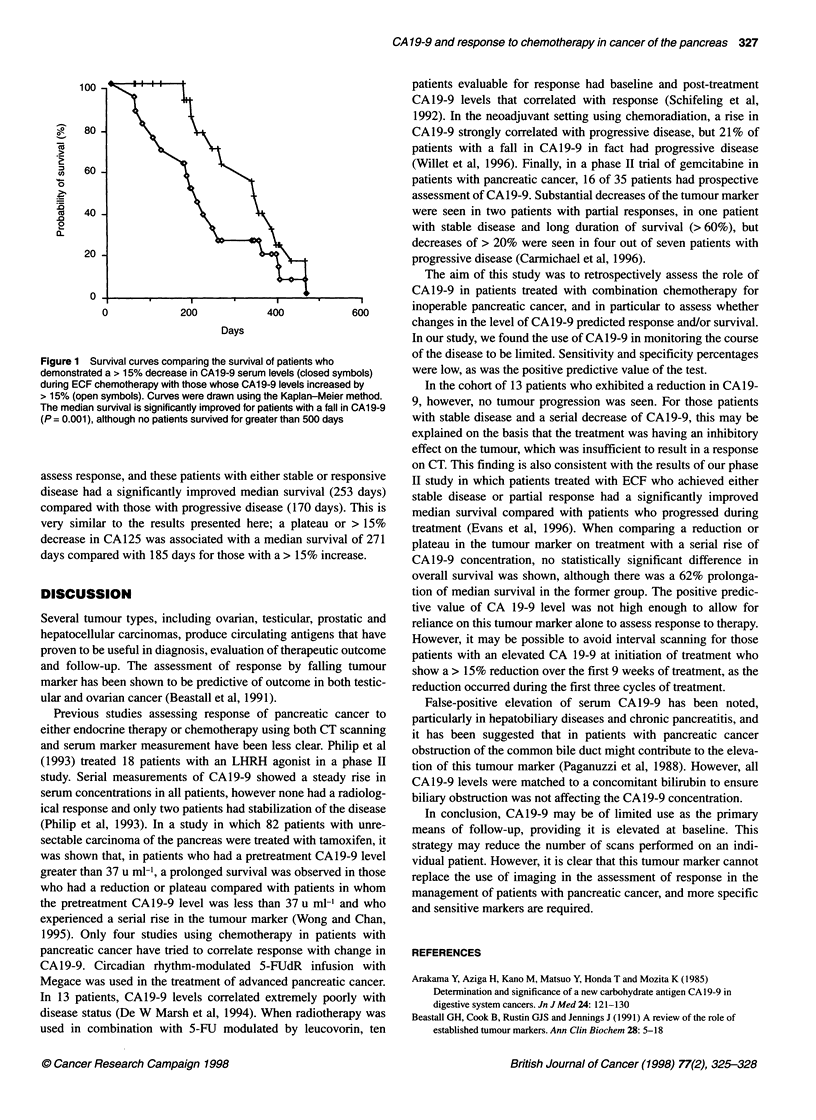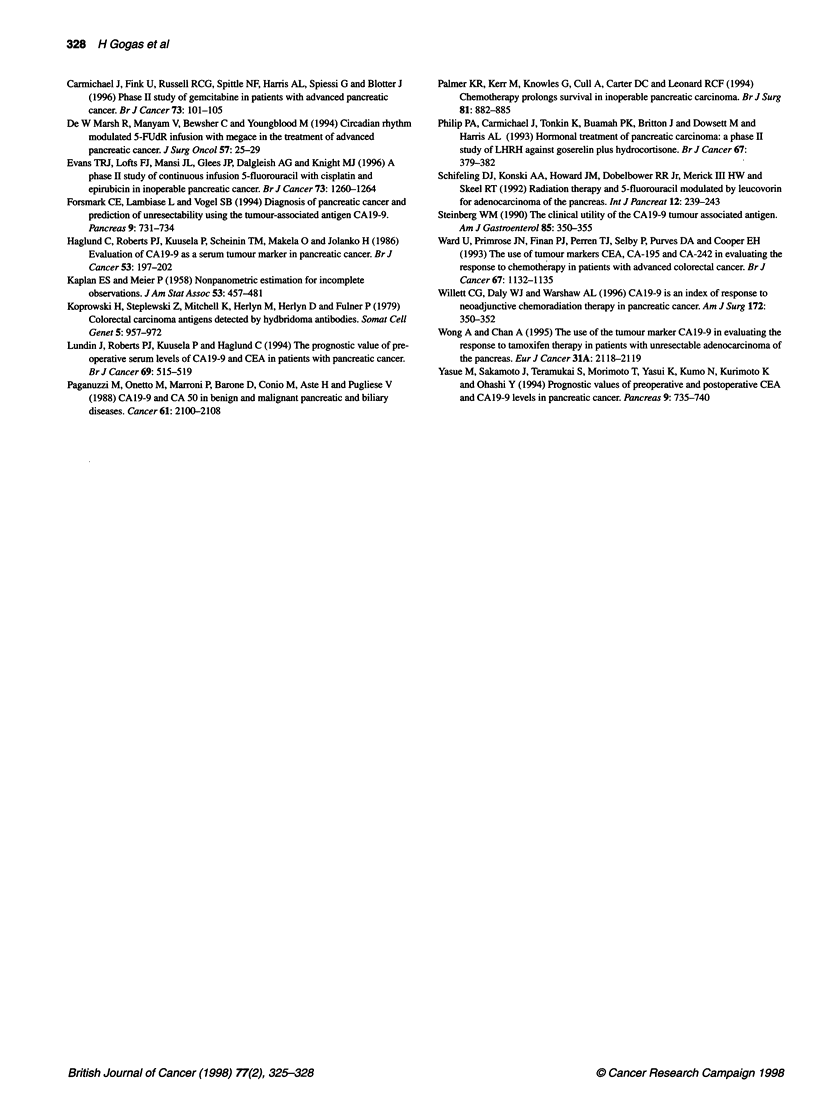# Are serial measurements of CA19-9 useful in predicting response to chemotherapy in patients with inoperable adenocarcinoma of the pancreas?

**DOI:** 10.1038/bjc.1998.50

**Published:** 1998

**Authors:** H. Gogas, F. J. Lofts, T. R. Evans, S. Daryanani, J. L. Mansi

**Affiliations:** Department of Oncology, St George's Hospital, London, UK.

## Abstract

Thirty-nine patients with inoperable adenocarcinoma of the pancreas were studied (27 male, 12 female; median age 60 years, range 39-75 years). All patients received chemotherapy with continuous infusion 5-fluorouracil with intravenous bolus epirubicin followed by cisplatin, repeated every 21 days for a total of six cycles and were evaluable for response. Serum CA19-9 concentrations were obtained at baseline and before each cycle. A rise or fall in the tumour marker was defined as a greater than 15% increase or decrease in the marker on two consecutive occasions 3 weeks apart. A plateau in the tumour marker was defined as a less than 15% decrease or increase on two occasions. Changes in marker expression were compared with serial computerized tomography scanning before treatment and after the third and sixth cycle of chemotherapy. Thirty-five of 39 patients had an elevated CA19-9 (87.9%). Thirteen (36.2%) exhibited a decrease, seven (19.4%) a plateau and 16 (44.4%) patients had a progressive rise in serum CA19-9. The sensitivity of CA19-9 was 67% for predicting a partial response and 86% for progressive disease. The median survival for the 13 patients exhibiting a reduction was 333 days, for the seven patients exhibiting a plateau 253 days and for those who had a progressive rise 185 days. The difference in median survival between the group of patients with > 15% decrease and those with > 15% increase of CA19-9 was significant (P = 0.001). In the cohort of patients who exhibited a reduction in CA19-9, no tumour progression was seen, and the reduction occurred during the first three cycles of treatment. Thus, interval scanning may be avoided in this group of patients.


British Journal of Cancer (1998) 77(2), 325–328
© 1998 Cancer Research Campaign

### Are serial measurements of CA19-9 useful in predicting response to chemotherapy in patients with inoperable adenocarcinoma of the pancreas?

**H Gogas, FJ Lofts, TRJ Evans, S Daryanani and JL Mansi**

Department of Oncology, Level 3, St James' Wing, St George's Hospital, London SW17 0QT, UK

**Summary** Thirty-nine patients with inoperable adenocarcinoma of the pancreas were studied (27 male, 12 female; median age 60 years, range 39–75 years). All patients received chemotherapy with continuous infusion 5-fluorouracil with intravenous bolus epirubicin followed by cisplatin, repeated every 21 days for a total of six cycles and were evaluable for response. Serum CA19-9 concentrations were obtained at baseline and before each cycle. A rise or fall in the tumour marker was defined as a greater than 15% increase or decrease in the marker on two consecutive occasions 3 weeks apart. A plateau in the tumour marker was defined as a less than 15% decrease or increase on two occasions. Changes in marker expression were compared with serial computerized tomography scanning before treatment and after the third and sixth cycle of chemotherapy. Thirty-five of 39 patients had an elevated CA19-9 (87.9%). Thirteen (36.2%) exhibited a decrease, seven (19.4%) a plateau and 16 (44.4%) patients had a progressive rise in serum CA19-9. The sensitivity of CA19-9 was 67% for predicting a partial response and 86% for progressive disease. The median survival for the 13 patients exhibiting a reduction was 333 days, for the seven patients exhibiting a plateau 253 days and for those who had a progressive rise 185 days. The difference in median survival between the group of patients with > 15% decrease and those with > 15% increase of CA19-9 was significant ($P = 0.001$). In the cohort of patients who exhibited a reduction in CA19-9, no tumour progression was seen, and the reduction occurred during the first three cycles of treatment. Thus, interval scanning may be avoided in this group of patients.

**Keywords:** CA19-9; pancreas; cancer chemotherapy; response

Patients with adenocarcinoma of the pancreas have a particularly poor survival with less than 1% alive 5 years from diagnosis. Although it has been shown that patients who receive chemotherapy tend to have a longer survival than a control group receiving no treatment, the disease is relatively chemoresistant (Palmer et al, 1994). The response rate, defined as a 50% reduction in tumour size, is lower than that seen with other cancers and assessment requires serial imaging, commonly using CT scanning. Evaluation by this means can be difficult because of the high proportion of fibrotic or inflammatory tissue relative to tumour, which translates into a low response rate. In addition, it is expensive and time-consuming.

Tumour markers represent a potentially simple and inexpensive method of monitoring response. CA19-9 is the carbohydrate antigen defined by monoclonal antibody 1116 NS 19-9 and is the sialylated Lewis$^a$ blood group antigen (Koprowski et al, 1979). Approximately 75% of patients with pancreatic carcinoma exhibit elevated levels of serum CA19-9 at diagnosis, and it has been shown to be more specific than carcinoembryonic antigen (CEA) (Haglund et al, 1986). CA19-9 levels greater than 100 u ml$^{-1}$ have a mean specificity of 98% for adenocarcinoma of the pancreas, and thus it is considered to be the standard serum marker in the management of this malignancy (Steinberg, 1990). Elevated preoperative levels have been found to correlate with a poor prognosis, and markedly high concentrations (> 1000 u ml$^{-1}$) predict for a low probability of surgical resection. Conversely, low preoperative levels predict a better survival (Forsmark et al, 1994; Yasue et al, 1994). An increase within 1 month of surgery is associated with a poorer outcome (Haglund et al, 1986; Yasue et al, 1994), whereas a fall predicts for a longer survival (Yasue et al, 1994). Interestingly, a normal or raised CA19-9 level does not appear to be independently predictive of survival when correlated with stage of disease, with no difference in survival being seen in patients with stage I or IV disease (Lundin et al, 1994).

We set out to determine whether changes in CA19-9 concentrations could be used to predict the response to chemotherapy in patients with pancreatic cancer, as determined by conventional WHO criteria of bidimensional diameter measurements of tumours on radiological imaging.

### PATIENTS AND METHODS

Thirty-nine patients referred for chemotherapy with inoperable pancreatic cancer were studied; 27 were male and 12 female. The median age was 60 years (range 39–75 years). Eighteen patients had metastatic disease and 21 locally advanced disease. All patients received chemotherapy with continuous infusion 5-fluorouracil (5-FU) at a dose of 200 mg m$^{-2}$ day$^{-1}$, with intravenous bolus epirubicin (50 mg m$^{-2}$) followed by cisplatin (60 mg m$^{-2}$) on day 1, repeated every 21 days for up to six cycles. All were evaluable for assessment of response. The results of our experience with the ECF (epirubicin, cisplatin, 5-FU) regimen in pancreatic cancer have been published previously (Evans et al, 1996).

*Received 29 November 1996*
*Revised 10 June 1997*
*Accepted 25 June 1997*

*Correspondence to:* JL Mansi, Department of Oncology, St George's Hospital, Blackshaw Road, London SW17 0QT, UK

Serum samples for tumour marker assessment were obtained at baseline and before each cycle thereafter from all patients. The serum CA19-9 concentration was measured using a commercially available solid-phase enzyme immunoassay (CIS, France). The normal value of < 37 u ml$^{-1}$ for CA19-9 was set by the manufacturers based on excluding approximately 99% of healthy controls. As obstruction of the common bile duct results in elevated CA19-9, bilirubin levels were assessed concomitantly with CA19-9 levels to ascertain that they were within normal limits (Paganuzzi et al, 1988). Patients who became jaundiced because of a blocked stent between cycles of chemotherapy were not excluded, as it has previously been shown that, in cases in which CA19-9 is abnormal because of biliary stasis, resolution of the blockage allows CA19-9 levels to return to the normal range within hours (Arakama et al, 1985).

A rise or fall in the tumour marker was defined as a greater than 15% increase or decrease in the marker on two consecutive occasions 3 weeks apart. A plateau in the tumour marker was defined as a less than 15% decrease or increase on two occasions. For the purposes of this study, a greater than 15% increase was considered to be 'positive' with regard to the detection of progressive disease and a greater than 15% decrease to be 'positive' for a response to treatment, as previously described (Ward et al, 1993; Wong and Chan, 1995). Tumours were assessed by computerized tomography (CT) scanning before treatment and after the third and sixth cycle of chemotherapy. Response evaluation was based on the WHO criteria.

Changes in marker expression were compared with serial CT scanning. Comparison of changes in tumour markers with CT in patients achieving an objective response or progressive disease were expressed in terms of sensitivity, specificity and a positive or negative predictive value. The following definitions apply:

$$\text{Sensitivity: } \frac{\text{true positive}}{(\text{true positive} + \text{false negative})} \times 100\%$$

$$\text{Specificity: } \frac{\text{true negative}}{(\text{true negative} + \text{false positive})} \times 100\%$$

$$\text{Positive predictive value: } \frac{\text{true positive}}{(\text{true positive} + \text{false positive})} \times 100\%$$

$$\text{Negative predictive value: } \frac{\text{true negative}}{(\text{true negative} + \text{false negative})} \times 100\%$$

Survival curves were drawn using the Kaplan–Meier method and analysed by the Wilcoxon rank test (Kaplan and Meier, 1958).

### RESULTS

Thirty-five of 39 patients treated with ECF (87.9%) had an elevated CA19-9 greater than 37 u ml$^{-1}$. Four patients had normal pretreatment levels. In three patients, CA19-9 remained at < 37 u ml$^{-1}$ during the whole course of treatment; one had a partial response, one stable disease and one progressive disease. It is known that approximately 5% of the population do not express the Lewis[a] antigen, which is in keeping with this observation. As these patients were non-contributory, they were excluded from further analysis. Of the 18 patients with metastatic disease, 94.4% had elevated CA19-9 serum levels at presentation compared with 85.7% with locally advanced tumours. Thirteen patients (36.2%) exhibited a decrease in serial measurement of serum CA19-9 levels while receiving ECF chemotherapy (in ten the decrease in tumour marker level was seen at the second cycle and in three at the third cycle). Seven (19.4%) patients showed a plateau, and the remaining 16 (44.4%) patients had a progressive rise in CA19-9. Corresponding bilirubin levels were within normal limits.

Comparison between the trend in the marker and the CT findings is shown in Table 1. The fall in CA19-9 was not sensitive in the prediction of partial response (67%) and the specificity was 69%, resulting in a positive predicative value of only 30% and a negative predictive value of 87%. Considering the prediction of progressive disease, the sensitivity of rising CA19-9 was 86%, but the positive predictive value was only 37%. It has been argued that CT assessment of metastatic lesions is more likely to demonstrate a reduction in bidimensional measurements, and thus correlation of changes in CA19-9 to CT changes was undertaken in the subgroup of patients with metastatic disease. Unfortunately, no partial remissions were seen in metastatic disease assessed either by CT or by fall in CA19-9 levels, and thus the sensitivity and positive predictive value for a fall in CA19-9 was 0%.

Median survival of the entire group of 39 patients was 251 days (range 54–459 days). The median survival for the 13 patients exhibiting a reduction in CA19-9 was 333 days. For the seven patients exhibiting a plateau, the median survival was 253 days and for those patients who had a progressive rise in CA19-9 was 185 days. There was no significant difference in the overall survival of patients with a reduction or plateau of CA19-9 or reduction alone compared with those with a serial rise in the tumour marker ($P$ = 0.1) (Figure 1). However, the difference in median survival of 185 days for the patients with a > 15% increase and 333 days for those with a > 15% decrease in tumour marker was significant ($P$ = 0.001) (Wilcoxon rank test). We have previously reported our experience using ECF in inoperable pancreatic cancer (Evans et al, 1996). In this report, CT scans were used to

**Table 1** Sensitivity, specificity and positive and negative predictive values of serial CA19-9 measurements in evaluating a partial response, stable or progressive disease as demonstrated by CT scanning

| | Partial response | Stable disease | Progressive disease |
|---|---|---|---|
| Sensitivity (%) | 4/6 (67) | 6/23 (26) | 6/7 (86) |
| Specificity (%) | 20/29 (69) | 12/16 (86) | 18/29 (67) |
| Positive predictive value (%) | 4/13 (30) | 6/7 (86) | 6/16 (37) |
| Negative predictive value (%) | 20/23 (87) | 12/29 (41) | 18/20 (90) |

 © Cancer Research Campaign 1998

**Figure 1** Survival curves comparing the survival of patients who demonstrated a > 15% decrease in CA19-9 serum levels (closed symbols) during ECF chemotherapy with those whose CA19-9 levels increased by > 15% (open symbols). Curves were drawn using the Kaplan–Meier method. The median survival is significantly improved for patients with a fall in CA19-9 ($P$ = 0.001), although no patients survived for greater than 500 days assess response, and these patients with either stable or responsive disease had a significantly improved median survival (253 days) compared with those with progressive disease (170 days). This is very similar to the results presented here; a plateau or > 15% decrease in CA125 was associated with a median survival of 271 days compared with 185 days for those with a > 15% increase.

### DISCUSSION

Several tumour types, including ovarian, testicular, prostatic and hepatocellular carcinomas, produce circulating antigens that have proven to be useful in diagnosis, evaluation of therapeutic outcome and follow-up. The assessment of response by falling tumour marker has been shown to be predictive of outcome in both testicular and ovarian cancer (Beastall et al, 1991).

Previous studies assessing response of pancreatic cancer to either endocrine therapy or chemotherapy using both CT scanning and serum marker measurement have been less clear. Philip et al (1993) treated 18 patients with an LHRH agonist in a phase II study. Serial measurements of CA19-9 showed a steady rise in serum concentrations in all patients, however none had a radiological response and only two patients had stabilization of the disease (Philip et al, 1993). In a study in which 82 patients with unresectable carcinoma of the pancreas were treated with tamoxifen, it was shown that, in patients who had a pretreatment CA19-9 level greater than 37 u $ml^{-1}$, a prolonged survival was observed in those who had a reduction or plateau compared with patients in whom the pretreatment CA19-9 level was less than 37 u $ml^{-1}$ and who experienced a serial rise in the tumour marker (Wong and Chan, 1995). Only four studies using chemotherapy in patients with pancreatic cancer have tried to correlate response with change in CA19-9. Circadian rhythm-modulated 5-FUdR infusion with Megace was used in the treatment of advanced pancreatic cancer. In 13 patients, CA19-9 levels correlated extremely poorly with disease status (De W Marsh et al, 1994). When radiotherapy was used in combination with 5-FU modulated by leucovorin, ten patients evaluable for response had baseline and post-treatment CA19-9 levels that correlated with response (Schifeling et al, 1992). In the neoadjuvant setting using chemoradiation, a rise in CA19-9 strongly correlated with progressive disease, but 21% of patients with a fall in CA19-9 in fact had progressive disease (Willet et al, 1996). Finally, in a phase II trial of gemcitabine in patients with pancreatic cancer, 16 of 35 patients had prospective assessment of CA19-9. Substantial decreases of the tumour marker were seen in two patients with partial responses, in one patient with stable disease and long duration of survival (> 60%), but decreases of > 20% were seen in four out of seven patients with progressive disease (Carmichael et al, 1996).

The aim of this study was to retrospectively assess the role of CA19-9 in patients treated with combination chemotherapy for inoperable pancreatic cancer, and in particular to assess whether changes in the level of CA19-9 predicted response and/or survival. In our study, we found the use of CA19-9 in monitoring the course of the disease to be limited. Sensitivity and specificity percentages were low, as was the positive predictive value of the test.

In the cohort of 13 patients who exhibited a reduction in CA19-9, however, no tumour progression was seen. For those patients with stable disease and a serial decrease of CA19-9, this may be explained on the basis that the treatment was having an inhibitory effect on the tumour, which was insufficient to result in a response on CT. This finding is also consistent with the results of our phase II study in which patients treated with ECF who achieved either stable disease or partial response had a significantly improved median survival compared with patients who progressed during treatment (Evans et al, 1996). When comparing a reduction or plateau in the tumour marker on treatment with a serial rise of CA19-9 concentration, no statistically significant difference in overall survival was shown, although there was a 62% prolongation of median survival in the former group. The positive predictive value of CA 19-9 level was not high enough to allow for reliance on this tumour marker alone to assess response to therapy. However, it may be possible to avoid interval scanning for those patients with an elevated CA 19-9 at initiation of treatment who show a > 15% reduction over the first 9 weeks of treatment, as the reduction occurred during the first three cycles of treatment.

False-positive elevation of serum CA19-9 has been noted, particularly in hepatobiliary diseases and chronic pancreatitis, and it has been suggested that in patients with pancreatic cancer obstruction of the common bile duct might contribute to the elevation of this tumour marker (Paganuzzi et al, 1988). However, all CA19-9 levels were matched to a concomitant bilirubin to ensure biliary obstruction was not affecting the CA19-9 concentration.

In conclusion, CA19-9 may be of limited use as the primary means of follow-up, providing it is elevated at baseline. This strategy may reduce the number of scans performed on an individual patient. However, it is clear that this tumour marker cannot replace the use of imaging in the assessment of response in the management of patients with pancreatic cancer, and more specific and sensitive markers are required.

### REFERENCES

Arakama Y, Aziga H, Kano M, Matsuo Y, Honda T and Mozita K (1985) Determination and significance of a new carbohydrate antigen CA19-9 in digestive system cancers. *Jn J Med* **24**: 121–130

Beastall GH, Cook B, Rustin GJS and Jennings J (1991) A review of the role of established tumour markers. *Ann Clin Biochem* **28**: 5–18

© Cancer Research Campaign 1998

Carmichael J, Fink U, Russell RCG, Spittle NF, Harris AL, Spiessi G and Blotter J (1996) Phase II study of gemcitabine in patients with advanced pancreatic cancer. *Br J Cancer* **73**: 101–105

De W Marsh R, Manyam V, Bewsher C and Youngblood M (1994) Circadian rhythm modulated 5-FUdR infusion with megace in the treatment of advanced pancreatic cancer. *J Surg Oncol* **57**: 25–29

Evans TRJ, Lofts FJ, Mansi JL, Glees JP, Dalgleish AG and Knight MJ (1996) A phase II study of continuous infusion 5-fluorouracil with cisplatin and epirubicin in inoperable pancreatic cancer. *Br J Cancer* **73**: 1260–1264

Forsmark CE, Lambiase L and Vogel SB (1994) Diagnosis of pancreatic cancer and prediction of unresectability using the tumour-associated antigen CA19-9. *Pancreas* **9**: 731–734

Haglund C, Roberts PJ, Kuusela P, Scheinin TM, Makela O and Jolanko H (1986) Evaluation of CA19-9 as a serum tumour marker in pancreatic cancer. *Br J Cancer* **53**: 197–202

Kaplan ES and Meier P (1958) Nonpanometric estimation for incomplete observations. *J Am Stat Assoc* **53**: 457–481

Koprowski H, Steplewski Z, Mitchell K, Herlyn M, Herlyn D and Fulner P (1979) Colorectal carcinoma antigens detected by hydbridoma antibodies. *Somat Cell Genet* **5**: 957–972

Lundin J, Roberts PJ, Kuusela P and Haglund C (1994) The prognostic value of pre-operative serum levels of CA19-9 and CEA in patients with pancreatic cancer. *Br J Cancer* **69**: 515–519

Paganuzzi M, Onetto M, Marroni P, Barone D, Conio M, Aste H and Pugliese V (1988) CA19-9 and CA 50 in benign and malignant pancreatic and biliary diseases. *Cancer* **61**: 2100–2108

Palmer KR, Kerr M, Knowles G, Cull A, Carter DC and Leonard RCF (1994) Chemotherapy prolongs survival in inoperable pancreatic carcinoma. *Br J Surg* **81**: 882–885

Philip PA, Carmichael J, Tonkin K, Buamah PK, Britton J and Dowsett M and Harris AL (1993) Hormonal treatment of pancreatic carcinoma: a phase II study of LHRH against goserelin plus hydrocortisone. *Br J Cancer* **67**: 379–382

Schifeling DJ, Konski AA, Howard JM, Dobelbower RR Jr, Merick III HW and Skeel RT (1992) Radiation therapy and 5-fluorouracil modulated by leucovorin for adenocarcinoma of the pancreas. *Int J Pancreat* **12**: 239–243

Steinberg WM (1990) The clinical utility of the CA19-9 tumour associated antigen. *Am J Gastroenterol* **85**: 350–355

Ward U, Primrose JN, Finan PJ, Perren TJ, Selby P, Purves DA and Cooper EH (1993) The use of tumour markers CEA, CA-195 and CA-242 in evaluating the response to chemotherapy in patients with advanced colorectal cancer. *Br J Cancer* **67**: 1132–1135

Willett CG, Daly WJ and Warshaw AL (1996) CA19-9 is an index of response to neoadjunctive chemoradiation therapy in pancreatic cancer. *Am J Surg* **172**: 350–352

Wong A and Chan A (1995) The use of the tumour marker CA19-9 in evaluating the response to tamoxifen therapy in patients with unresectable adenocarcinoma of the pancreas. *Eur J Cancer* **31A**: 2118–2119

Yasue M, Sakamoto J, Teramukai S, Morimoto T, Yasui K, Kumo N, Kurimoto K and Ohashi Y (1994) Prognostic values of preoperative and postoperative CEA and CA19-9 levels in pancreatic cancer. *Pancreas* **9**: 735–740

*© Cancer Research Campaign 1998*